# Safety, Efficiency, and Mental Workload of Predictive Display in Simulated Teledriving

**DOI:** 10.3390/s26010221

**Published:** 2025-12-29

**Authors:** Oren Musicant, Alexander Kuperman, Rotem Barachman

**Affiliations:** 1 Engineering Faculty, Ariel University, 3 Kiryat Hamada St., Ariel 4070000, Israel; alexandrk@ariel.ac.il; 2 Faculty of Humanities and Social Sciences, Ariel University, 3 Kiryat Hamada St., Ariel 4070000, Israel; rotem.barachma@msmail.ariel.ac.il

**Keywords:** remote driving, predictive display, communication time delay, driver performance

## Abstract

Vehicle remote driving services are increasingly used in urban settings. Yet, vehicle-operator communication time delays may pose a challenge for teleoperators in maintaining safety and efficiency. The purpose of this study was to examine whether Predictive Displays (PDs), which show the vehicle’s predicted real-time position, improve performance, safety, and mental workload under moderate time delays typical of 4G/5G networks. Twenty-nine participants drove a simulated urban route containing pedestrian crossings, overtaking, gap acceptance, and traffic light challenges under three conditions: 50 ms delay (baseline), 150 ms delay without PD, and 150 ms delay with PD. We analyzed the counts of crashes and navigation errors, task completion times, and the probability and intensity of braking and steering events, as well as self-reports of workload and usability. Results indicate that though descriptive trends indicated slightly sharper steering and braking under the 150 ms time delay conditions, the 150 ms time delay did not significantly degrade performance or increase workload compared with the 50 ms baseline. In addition, the PD neither improved performance nor reduced workload. Overall, participants demonstrated tolerance to typical 4G/5G network time delays, leaving little room for improvement rendering the necessitating of PDs.

## 1. Introduction

Teleoperation, the remote control of vehicles, is helpful in multiple use cases. For example, delivering a shared vehicle from one person to another or remote taxi services [1]. In addition, teleoperation can be useful in edge-case scenarios where autonomous vehicles do not perform as well as human drivers. For example, when it is necessary to violate traffic rules (e.g., the road is blocked and one must cross a separation line to proceed) or when a police officer is regulating traffic by hand gestures [2].

Nowadays, commercial operators deploy teleoperation services in real-world mobility services. For instance, Cruise has confirmed that its robotaxis rely on human teleoperators’ assistance approximately every 4–5 miles [3], Vay has launched remote driving operations in Las Vegas [4], and Waymo has detailed the design of its fifth-generation driver to enable remote support [5]. These services are especially active in urban environments, where complex, edge-case scenarios frequently require human judgment. As such, any system intended to support teleoperation, such as predictive displays, must be evaluated under realistic conditions that mirror this deployment context.

In teleoperation, one of the key challenges teledrivers (hereafter TEDs or operators) face is the time delay between the vehicle’s movements and the feedback the operator receives. Time delay can significantly impact TED’s ability to respond in real time to changes in the environment, especially in dynamic urban settings where hazards and obstacles may appear suddenly. High delay can lead to delayed reactions, which in turn can compromise both the safety and efficiency of teleoperation tasks [1,6].

A promising approach to mitigate delay effects is the use of Predictive Displays (PDs). These systems estimate the vehicle’s true location by accounting for communication delay, speed, and trajectory, and then visualize this estimation on the operator’s screen. By showing where the vehicle actually is, rather than where it appears after the delay, PDs help TEDs better align their control inputs with real-world conditions. This bridging of the delay gap enhances situational awareness, thereby improving both safety and operational efficiency in dynamic, complex traffic environments.

## 2. Related Work

### 2.1. The Human Factors Challenges of Teledrivers (TEDs)

Teledriving of autonomous vehicles introduces a distinct set of human factors challenges beyond those faced by regular drivers. One prominent issue is the limited situational awareness experienced by Teledrivers (TEDs), who must rely on indirect and often degraded sensor-based visual input instead of direct perception [7,8]. This reduced sensory fidelity compromises hazard perception [9,10], especially during takeover requests, when operators must regain control under time pressure and in unfamiliar contexts [11,12]. Compounding this, is the challenge of switching between AVs, which requires rapid adaptation to varying vehicle interfaces, operational goals, and traffic scenes, a task not typically demanded of regular drivers [13].

In addition, communication time delays introduce significant technological demands that can impair real-time control, leading TEDs to either overcompensate or adopt inefficient “move and wait” strategies [8,14]. The absence of haptic feedback and the inability to physically experience vehicle dynamics further hinder TEDs’ ability to judge acceleration, braking, and road conditions [15,16]. These deficits may be exacerbated by a lack of embodiment; TEDs may not develop the same sense of ownership or physical connection to the vehicle, raising concerns about moral detachment and reduced accountability [17,18]. Overall, these challenges underscore the need for targeted technological and organizational solutions tailored specifically to TEDs. The current work focuses only on the communication time delay challenge and one mitigation strategy (PDs). The remainder of this review will therefore elaborate on the relevant studies. Readers interested in a broader overview of teledriving challenges and corresponding mitigation strategies are encouraged to consult the review by Meir Grimberg and Musicant [19].

### 2.2. Remote Driving Under Time Delay

Time delay in teleoperation is the time elapsed between an event occurring in the remote environment (or control station, e.g., the operator issuing a steering or braking command) and its perception by the TED (remote vehicle), or vice versa. It results from bidirectional transmission over wireless networks, where video streams from the vehicle to the operator, and control signals are sent back [2,20,21].

Time delay impairs TEDs’ abilities in several ways: First, it disrupts spatial orientation, a cognitive process, which integrates direction and speed over time [22]. When feedback is delayed, the operator’s estimate of the vehicle position and heading becomes misaligned with the vehicle’s true state, especially during continuous directional change such as curve negotiation [23]. Next, even when TEDs remain oriented, due to the time delay, their ability to generate timely response is still limited. In addition, time delay increases mental workload. TEDs must mentally compensate for outdated feedback and delayed action execution. This added complexity elevates task demands and degrades overall performance, especially in dynamic environments with multiple road users [14,21,23,24].

These human factor risks persist even at short delays (e.g., 150–250 ms), which characterize modern low-latency 5G networks [2]. Recent simulation studies have shown that even at these sub-second delays, driver performance and safety-related kinematics, such as steering or maintaining headway, may degrade [14,25]. Therefore, countermeasures such as predictive displays have been proposed to mitigate the functional disconnect between TED input and system response.

### 2.3. Predictive Displays (PDs)

A predictive display (PD) is a visual aid designed to mitigate the effects of time delay in teleoperation by presenting the TED with a real-time prediction of the remote vehicle’s state. Rather than displaying only delayed sensor or video feedback, a PD uses motion models and control inputs to estimate and visualize how the vehicle is expected to move in the immediate future, typically over the next few hundred milliseconds (ms). By compensating for delay-induced lag in perception, PDs help maintain spatial awareness and control continuity, potentially improving safety and task performance under time delay conditions. The literature indicates that PDs can partially mitigate the effects of delays on TEDs’ performance. Table 1 summarizes several examples. The data in the table provide insight into the potential benefits of PDs as a mitigation strategy, the specific time delays examined, and the performance and safety indices used to evaluate their effectiveness.

#### 2.3.1. The Benefits of Predictive Displays

Brudnak [26] conducted a simulation-based study to evaluate the impact of a PD on the teleoperation of an unmanned ground vehicle under high-delay conditions. Three conditions were tested: (1) no delay and no prediction, (2) 500 ms delay without PD, and (3) 500 ms delay with a PD. The experiment showed that a 500 ms delay reduced speed by 30%, and increased path deviation and heading error by 148% and 180%, respectively. Adding a PD significantly improved performance relative to the delay-only condition; speed increased by 29%, path deviation decreased by 35%, and heading error decreased by 42%.

Prakash et al. [27] examined the use of a PD to improve vehicle teleoperation under variable network delays (70–150 ms). Participants controlled a remotely operated vehicle at 10 km/h, completing two laps with cornering and double-lane change maneuvers to simulate realistic driving tasks. Two conditions were tested: (1) baseline teleoperation with delay and no PD, and (2) teleoperation with a PD that processed the video feed to forecast the vehicle’s expected position once operator commands were executed. The PD significantly reduced path deviation and improved driver control and confidence, demonstrating its practical effectiveness for teleoperation in real-world network conditions.

Dybvik et al. [21] examined the impact of PDs on task performance and cognitive workload during a precision navigation task, where participants maneuvered a vehicle into a marked target zone. Each participant completed the task under three conditions: (1) 700 ms delay without PD, (2) 700 ms delay with PD, and (3) baseline with 250 ms inherent delay. Using the PD improved task performance by 20%, while subjective workload (NASA-TLX) showed no significant reduction, though participants reported higher perceived performance and lower frustration. Interestingly, self-identified gamers achieved nearly twice the improvement, suggesting that PDs can effectively offset communication delays in remote operations.

The studies we reviewed and summarized in Table 1 consistently demonstrated improvements in operator performance when using a PD. The implementation of PDs led to reduced path deviation, increased task accuracy, and, in the case of Brudnak [26], increased driving speed.

#### 2.3.2. Time Delays in PD Studies

The selection of time delays varies across studies, reflecting different research aims and operational assumptions. Brudnak [26] conducted a simulation-based study using a fixed 500 ms delay representing a worst-case scenario, as delays above 200 ms are known to impair operator performance and induce a “move and wait” control strategy. Prakash et al. [27] adopted a variable delay range of 70–150 ms based on round-trip delay measurements collected during actual urban teleoperation trials using 4G LTE communication. Dybvik et al. [21] used a fixed 700 ms delay, citing prior findings that operator performance declines markedly within the 500–1000 ms range. Similarly, Davis et al. [20] applied a 700 ms delay derived from Army test bed operations, where delays commonly ranged from 200 ms to over 1000 ms. In both latter studies, 700 ms represented a demanding condition under which mitigation strategies like PDs could be effectively evaluated.

Overall, these studies illustrate three main rationales for delay selection: testing extreme conditions to probe mitigation limits [26]; adopting empirically measured real-world communication delays [27]; and focusing on delay levels known to impair teleoperator performance [20,21]. We note that only one study [27] focused real-world communication delays (70–150 ms) rather on the extreme time delays. More studies are needed per such time delay, perhaps with additional driving challenges (e.g., interaction with other road users) in addition to the curved driving scenarios studied by Prakash et al. [27].

#### 2.3.3. Driving Tasks

Previous PD studies have generally relied on simplified or constrained driving scenarios, such as lane-keeping on straight or curved segments [26,29], low-speed cornering [27], or single-target navigation tasks focused primarily on motion tracking under delayed feedback. These tasks were typically performed in rural environments, obstacle courses, or closed circuits with minimal interaction with dynamic road users. As a result, they required little beyond continuous control skills and provided limited opportunity to assess delay-sensitive decision-making such as overtaking, judging braking intensity at changing traffic lights, or responding to pedestrians. Real-world implementations (e.g., [3,4,5]) operate in dense, signalized environments with complex infrastructure and unpredictable road users, conditions that amplify the challenges of remote driving under time delay. It is therefore essential to evaluate PDs in ecologically valid urban scenarios, where operators are required not only for motion tracking abilities, but also to recruit other skills (such as hazard perception and spatial rotation) and engage decision-making mechanisms (e.g., increased defensive driving) that were rarely required in simpler scenarios.

#### 2.3.4. Indices Used in PD Studies

Performance measures used in PD studies focused primarily on basic vehicle control and maneuvering. These included lane-keeping accuracy [20,26,27,29], driving speed [20,26], and mental workload [20,29].

Notably, the PD studies we reviewed largely omitted analyses of undesired driving events, such as intense steering and braking. Such analysis was conducted in time delay studies (without the aid of a human–machine interface). For instance, Musicant, Botzer, and Shoval [14] reported an increase in steering events (modeled through lateral acceleration) as time delay increased from 50 ms to 150 ms and more prominently when time delay increased to 250 ms on a number of challenges in interurban road, not only in curve driving but also in response to lead vehicle sudden stop and when interacting with outer vehicles; for example, the probability of intense braking events (but not steering) increased when facing a roadblock. Neumeier et al. [25] also documented increases in steering (steering wheel angle) and acceleration under specific driving challenges. These extreme events are therefore a sensitive marker of delay-related perceptual–motor misalignment. In parallel, research found a correlation between the occurrence of such events and crashes [30,31,32,33], further establishing their use as safety surrogates. It is therefore interesting to study how PD can mitigate the occurrence of such events.

Regarding self-reported measures, the current literature does not yet account for important indices, such as the usability of predictive interfaces, and does not use standardized tools such as PSSUQ, indicating that important aspects of user satisfaction remain underexplored.

## 3. The Current Study-Research Assumptions and Hypotheses

In this study, we extend prior work by examining PDs in a simulated urban environment that more closely reflects current teleoperation practice. We hypothesize that increasing the communication time delay from a low baseline (50 ms) to 150 ms will degrade performance, as reflected in longer task completion times, more navigation errors, and more intense braking and steering events. Most previous studies examined much higher delays, using performance degradation as a test bed for PD usefulness. In contrast, we aim to quantify the contribution of PD at more realistic, modest levels of performance degradation, which better reflect real teleoperation conditions. The 150 ms delay level is comparable only to that of Prakash et al. [27].

Second, we hypothesize that, even with a realistic (rather than intentionally high) 150 ms time delay, PD may still improve performance. Urban driving challenges partly rely on the operator’s ability to perform continuous motion tracking and anticipate future vehicle movement, functions that PD is designed to support. At the same time, these challenges also depend on higher-level processes related to situational awareness and decision-making, such as deciding whether to overtake, yield to pedestrians, or stop or proceed when a traffic light changes. We aim to explore whether PD benefits performance and safety in complex urban scenarios that require more than pure motion control to be managed successfully.

Finally, while time delay studies used performance measures related to the occurrence of intense driving events (such as steering and braking), these measures are currently lacking in PD studies. We aim to study the effect of the PD interface on the occurrence of intense driving events. In addition to the objective performance measures and workload, we also assess perceived system usability to better understand whether operators are likely to adopt the PD interface in practice.

## 4. Methods

### 4.1. Participants

The study’s participants consisted of 29 students (21 females) volunteering to serve as TEDs in our study. The mean age was 25 (SD = 2), and the mean driving experience was 7.5 years, (SD = 2.5). The study was approved by the university ethics committee. All participants provided written informed consent before the experiment.

### 4.2. Design

We adopted a three-condition design: low time delay (50 ms) without PD (“50 ms PD Off”), high delay without PD (“150 ms PD Off”), and high delay with PD mitigation (“150 ms PD On”). This structure follows leading PD studies (e.g., [21,26,27]), in which PDs serve as compensatory tools for communication delays. Using this design, we investigated dependent variables from three categories: (1) Efficiency, which included task completion times for the full driving route and each embedded challenge, as well as the probability of navigation errors. Participants were instructed to follow directional signs; errors at predefined decision points (Table 2, Figure 2) led to simulation termination. (2) Safety, which included collision rates, steering intensity (lateral acceleration, m/s^2^), and maximal braking intensity (negative longitudinal acceleration, m/s^2^). (3) Driver state, assessed via subjective mental workload ratings. All dependent measures were analyzed as a function of delay condition (150 ms vs. 50 ms) and predictive display status (On vs. Off).

### 4.3. Apparatus and Materials

#### 4.3.1. Questionnaires

We used several questionnaires before, during, and after the experiment. Before arriving at the laboratory, participants reported their age, gender, and driving experience via a demographic questionnaire. At the end of each driving session, participants completed the NASA Task Load Index (NASA-TLX [34]), rating their subjective experience across six dimensions: mental demand, physical demand, temporal demand, performance, effort, and frustration, using a 9-point Likert scale. Finally, after the session that included the predictive display, participants completed the Post-Study System Usability Questionnaire (PSSUQ), using a 5-point Likert scale where higher scores indicated more favorable perceptions. The PSSUQ was administered only following the PD On condition, as it is designed to assess perceived usability of an actively used system. Since no assistive system was present in the baseline or delay-only conditions, applying the questionnaire in those cases would not have yielded meaningful or interpretable responses. The PSSUQ focused specifically on the usability, information quality, and usefulness of the predictive display as experienced by participants during teleoperation under delay.

#### 4.3.2. Driving Simulator

We utilized the CARLA simulator [35], to conduct simulations of the driving task. The CARLA simulation environment incorporates various elements, such as traffic lights, traffic signs, and other road users, allowing developers of autonomous vehicles to effectively test and train their driving algorithms. In this study, we focused on evaluating the performance of TEDs; while the ego vehicle (the vehicle being teleoperated by the participant) was not autonomous, the simulator managed other elements, including the behavior of surrounding road users.

**Figure 1:**
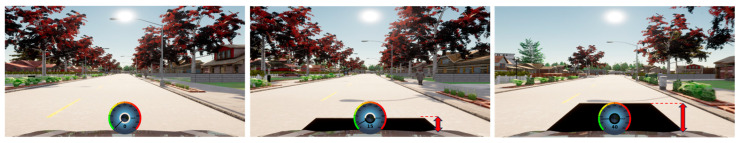
Predictive display operation.

To facilitate the operation of the ego vehicle by a human, we connected acceleration and braking pedals along with a force feedback steering wheel (Logitech G920, San Jose, CA, USA) to the simulator. The simulator setup included a fixed-base configuration with three 27-inch monitors, providing a forward field of view of 135 degrees (see Figure 1). The camera of the ego vehicle was mounted at a height of 1.63 m. The camera was also tilted at a pitch angle of 7.5 degrees, ensuring a realistic perspective for the simulation (see Figure 1).

To simulate time delays, we introduced fixed offsets between the teleoperator’s commands and the vehicle’s responses. In the 150 ms condition, for example, the vehicle began to decelerate 150 ms after the operator applied the brakes. In real teleoperation, overall delay reflects both uplink (vehicle to operator) and downlink (operator to vehicle) transmission. In simulator studies, however, delaying one direction yields comparable perceptual effects, as the operator perceives the vehicle responding after the total delay regardless of its source. Similar implementations follow the same rationale [14,21,36].

The PD estimated the vehicle’s near-future position using a constant-velocity extrapolation model. The extrapolated position, corresponds to the vehicle’s expected state after 150 ms. The predictive overlay appears as a black extension of the ego vehicle, representing its estimated position after 150 ms.

Each participant completed the task under two delay conditions: 150 ms and 50 ms. We tested the 150 ms condition twice, once with a predictive display and once without it. Figure 1 illustrates the predictive display function. The bottom panel displays screen captures of the predictive display in operation, while the top panel shows screen captures when the predictive display is not active.

#### 4.3.3. Carry-Over Effects

**Figure 2:**
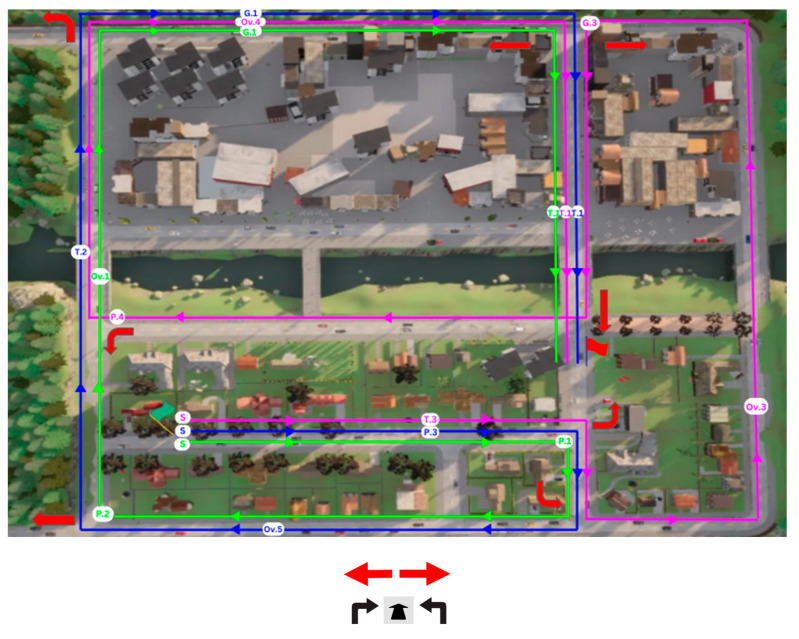
A map of the simulated route and significant points along it. Red arrows indicate locations where navigation errors sometimes occurred because participants did not respond correctly to the direction signs (black sights that appeared on the display of the participant). These signs are depicted at the bottom of the figure.

To mitigate potential carry-over effects, where a participant’s experience in one experimental condition influences behavior in subsequent conditions, studies commonly use randomization and washout periods [37]. In the present study, the order of conditions was randomized, and a brief calming meditation video was shown between runs as a washout period [38], promoting mental reset and more reliable responses across conditions. In addition to these steps, we designed three distinct simulator tracks, each presenting the driving challenges in a different sequence (Table 1, Figure 2). Track C was added in the middle of the study (applied for 15 out of 29 participants). The order of the tracks was also randomized, creating various combinations of track and experimental conditions. Furthermore, for each of the four driving challenges (Table 2), we created challenge variants (e.g., gap acceptance for trucks A and C was different), yielding a total of 12 variants across the driving challenges. These steps were taken to prevent participants from learning the locations or specific details of the challenges (e.g., the direction from which the pedestrian attempted to cross).

#### 4.3.4. Driving Challenges

Table 2 lists the driving challenges. These included turning, overtaking, and pedestrian crossing. Figure 2 illustrates the driving route with the driving challenges designated by corresponding letters (e.g., P for pedestrian) and numbers. The route length for each scenario type was approximately 1000 m on a simulated urban road with two lanes, and the participants drove on it in simulated daylight and clear weather conditions.

We note that in the analysis (subsequently described) we removed a short section of approximately 100 m for track B (total 2000 m). The removed segment, due to technical irregularities in the simulation, was not identical across all experimental conditions.

### 4.4. Procedure

After signing an informed consent form, participants were invited to participate in our driving simulator study; three simulated driving sessions were in an urban environment and three were in a highway environment. This study focuses on the three urban simulation runs. Upon arrival, the participants received detailed information regarding the procedure. They were instructed to drive as they normally would in real road conditions (e.g., obeying traffic rules, following speed limits, and keeping a safe headway). All participants completed one training session of approximately six minutes, followed by the experimental sessions (details in subsequent results section). The order of the experimental conditions (50 ms PD Off, 150 ms PD Off, 150 ms PD On) were counterbalanced. At the end of each session, participants completed the NASA-TLX questionnaire. After the PD On session, participants completed the PSSUQ.

### 4.5. Statistical Analyses

#### 4.5.1. Crashes and Navigation Errors

We estimated the probability of crash and navigation error using two separate mixed-effects logistic regression models. In each model, the binary outcome variable was coded as 1 if the trial ended in a crash (for the crash model) or a navigation error (for the error model), and 0 otherwise. The fixed-effects structure comprised two dummy variables contrasting the 150 ms delay conditions against the 50 ms baseline: one representing the trial with a 150 ms delay and PD off ($D_{i,150ms,PD\;Off}$), and the other representing the trial with a 150 ms delay and PD on ($D_{i,150ms,PD\;On}$). The following is the formal description of the model for crashes:

(1) $$Logit\;(P_{i,crash})=\beta_{0}+\beta_{1}D_{i,150ms,PD\;Off}+\beta_{2}D_{i,150ms,PD\;On}+\beta_{3}{Track\;B}_{i}+\beta_{4}{Track\;C}_{i}+bi$$

where $\beta_{0}$ represents the Lan (odds) of a crash under the baseline condition (50 ms delay, PD off). The coefficients $\beta_{1}$ and $\beta_{2}$ quantify the change in log-odds for the two 150 ms delay conditions relative to the baseline. Driving track was included as a categorical control variable, with Track A as the reference category and dummy variables for Tracks B and C. A random intercept bi ∼ N(0, σ) was included to account for participant-level variability. An identical model structure was used for the probability of navigation errors, replacing the outcome variable accordingly.

#### 4.5.2. Intensity of Braking and Steering Events

The intensity of braking and steering, two key measures of driving safety (see Section 2.3.4), is often estimated by their longitudinal or lateral acceleration (in absolute values), respectively. We explored the relationship between the experimental condition and the intensity of braking and steering events with two approaches. The first approach was estimating the probability of a braking or steering event for a range of intensity thresholds between 3 and 6.0 m/s^2^ in increments of 0.2 m/s^2^ (see [39,40], for additional examples of threshold-dependent analyses). The analysis was based on a logistic regression model:

(2) $$Ln\frac{E_{i}\left(Y_{g>T}\right)}{N-E_{i}\left(Y_{g>T}\right)}=\beta_{0,T}+\beta_{1,T}D_{i,150ms,PD\;Off}+\beta_{2,T}D_{i,150ms,PD\;On}+\beta_{3,T}{Track\;B}_{i}+\beta_{4,T}{Track\;C}_{i}+b_{i}$$

where E(Yg>T) is the expected count of braking (or steering) events that were more intense than T longitudinal (or lateral for steering) deceleration (m/s^2^), out of the total count (denoted by N) of braking (steering) events. $\beta_{0,T}$ is the intercept, representing the logit proportion of braking (or steering) events with at least T intensity with a 50 ms delay (the reference group). $D_{i,150ms,PD\;Off}\;and\;D_{i,150ms,PD\;On}$ are dummy variables for the experimental conditions, and $\beta_{1,T}$ and $\beta_{2,T}$ are the corresponding coefficients. Once again, we control for the possible effect of the specific track (A, B, or C) by introducing dummy variables (and corresponding coefficients) for the driving tracks (B and C). Finally, the term bi is a random effect parameter fitted for participant i, where bi is assumed to be normally distributed ($b_{i}$ ∼ N(0, σ)).

The second approach is for assessing how experimental conditions influenced peak braking and steering intensities across the driving challenges (see Table 2 and Figure 2). We fitted a linear mixed-effects model predicting the Lan-transformed maximum intensity per each challenge. The fixed-effects structure comprised the driving challenge (see Table 2), and the driving challenge interaction with the experimental condition, allowing condition effects to vary across challenges. The participant was specified as a random intercept to account for repeated measures. Here, we focused on challenges completed without interference from a collision or navigational error.

#### 4.5.3. Driving Challenges Completion Time

We analyzed the time it took participants to complete the driving challenges. As in the analysis on maximal braking and steering intensity, we used a mixed-effects model. We estimated the natural logarithm of the time to complete driving challenges as a function of the driving challenge (fixed effect), the challenge-by-condition interaction, allowing condition effects to vary across challenges, the specific track, and the participant (random effect). Here again, for each participant, we used data entries for all challenges (pedestrian crossings, overtaking…) when the driving task was completed, and fewer challenges if a crash (or a navigational error) occurred.

#### 4.5.4. Mental Workload

To assess the influence of the predictive display on mental workload, we fitted a single linear mixed-effects model to the NASA-TLX scores. The model specified separate baseline levels for each NASA-TLX subscale by removing the global intercept and including subscale as a fixed effect. The effect of the experimental condition (50 ms, 150 ms PD Off, 150 ms PD On) was modeled through the condition-by-subscale interaction, allowing condition effects to vary across subscales. The simulation track was included as a fixed effect to control for potential differences attributable to the track. Participants were entered as a random intercept to account for repeated measurements.

## 5. Results

The results are presented in three parts. First, we present the findings on driving efficiency according to task completion times and navigational errors. Second, we present the findings on safety according to crash occurrences and braking and steering intensity. Lastly, we present the results on the mental workload scores on the NASA-TLX.

### 5.1. Efficiency

#### 5.1.1. Navigation Errors

Participants were instructed to follow directional signs, but could err in taking the wrong turn (Figure 2). Descriptively, three navigation errors occurred in the 50 ms PD Off condition, five in the 150 ms PD Off, and two in the 150 ms PD On condition. Fisher’s exact test indicated that these differences were not statistically significant (*p* = 0.4). To account for track and participant variability, a logistic mixed-effects model with random intercepts for participants was fitted. Compared with the 50 ms PD Off baseline, the odds of error increased under 150 ms PD Off (OR = 2.1, CI: 0.3–12.4) and decreased under 150 ms PD On (OR = 0.5, CI: 0.1–4.0). A direct comparison between the two high-delay conditions showed that activating the PD reduced error odds by approximately 80% (OR = 0.2, CI: 0.03–1.8), though this effect was not statistically significant.

#### 5.1.2. Completion Times

**Figure 3:**
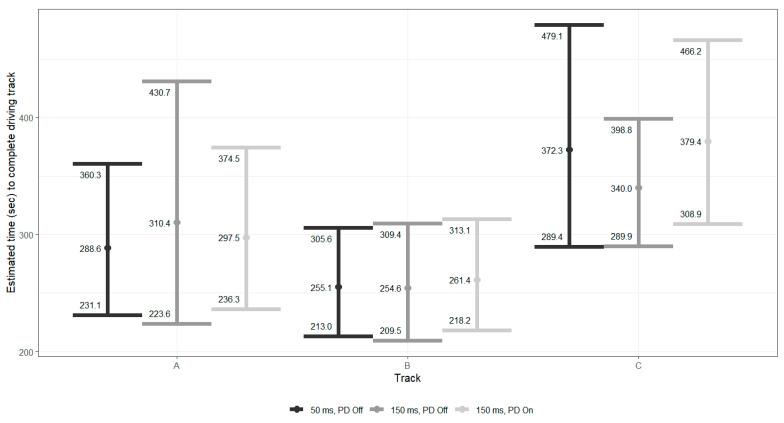
Mean completion times (±95% CI) for each driving track under the three experimental conditions.

The completion time for each driving track was unaffected by either increased delay or the activation of the PD as all 95% confidence intervals overlapped substantially, and no pairwise contrast reached statistical significance (all *p* > 0.05). Figure 3 details the estimated (and CI) completion times by driving tracks. The results indicate that neither increasing delay from 50 ms to 150 ms nor adding the PD produced a statistically reliable change in driving performance as measured by time to complete the track.

**Figure 4:**
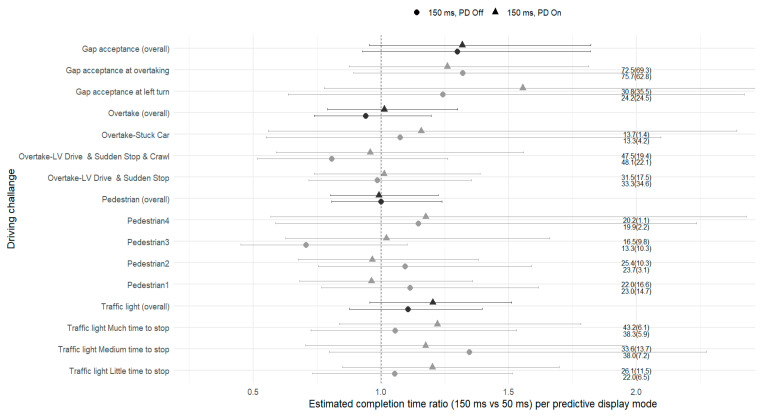
Estimated completion time ratios (150 ms vs. 50 ms) by driving challenge and predictive display condition (● PD Off, ▲ PD On). Error bars indicate 95% confidence intervals from the mixed-effects model controlling for repeated measures. Gray text next to each point shows the observed mean ± SD completion time for that challenge. Black symbols represent aggregate estimates across challenges. The dashed vertical line at 1.0 marks equal performance relative to the 50 ms baseline.

To examine whether PDs influenced the time required to complete individual driving challenges, we analyzed completion time ratios (150 ms/50 ms) with and without the display. Figure 4 presents estimated completion time with 95% confidence intervals derived from a mixed-effects model accounting for tracks and for repeated measures across participants (see Section 4).

None of the confidence intervals excluded 1.0, indicating that neither the increase in time delay nor activation of the PD produced statistically significant changes in task duration.

For the PD effect (150 ms PD On vs. 150 ms PD Off), completion times were similar across most challenge types. Aggregate ratios differed only slightly between display conditions, with none of the confidence intervals excluding 1.0. For the traffic light challenges, aggregate ratios were 1.1 (CI: 0.9–1.4) without the display and 1.2 (CI: 1.0–1.5) with it. The direct comparison between display modes yielded a ratio-of-ratios of 1.1 (CI: 0.9–1.4, *p* = 0.41), confirming the absence of a statistically significant difference. At the segment level, all three traffic light scenarios (little, medium, and much time to stop) produced ratios close to or slightly above 1.0 under PD On.

Pedestrian challenges aggregated near 1.0 (CI: 0.8–1.2) under both display conditions. Individual segments varied narrowly around unity, with all four pedestrian challenges showing near-identical mean times. Overtake challenges showed ratios of 0.9 (CI: 0.7–1.2) for PD Off and 1.0 (CI: 0.8–1.3) for PD On (ratio-of-ratios = 1.1 (CI: 0.8–1.4)). Segment values for Overtake–LV Drive and Sudden Stop, Overtake–LV Drive and Sudden Stop and Crawl, and Overtake–Stuck Car were all close to 1.0. In the gap acceptance category, aggregate ratios were 1.3 (CI: 0.9–1.8) without the display and 1.3 (CI: 1.0–1.8) with the display (ratio-of-ratios = 1.0 (CI: 0.7–1.5)). Segment level results for Gap acceptance at left turn and Gap acceptance at overtaking showed similar values, with wide confidence intervals encompassing 1.0.

When looking at the effect of the delay (150 ms PD Off vs. 50 ms PD Off), completion times remained largely stable. Aggregate ratios ranged between 0.9 and 1.3, and all confidence intervals included 1.0, indicating that a moderate 150 ms delay alone did not significantly affect performance. Descriptively, some challenges, especially the traffic light and gap acceptance tasks, showed ratios slightly above 1.0, whereas others clustered more closely around 1.0. However, none of these directional differences reached statistical significance.

### 5.2. Driving Safety

#### 5.2.1. Crashes

Crash occurrences were minimal across all conditions, with zero crashes recorded under the 50 ms delay and three crashes under each of the 150 ms delay conditions (with and without the predictive display). Due to the limited number of crashes, no statistical significance was found (Fisher test, *p* = 0.2).

#### 5.2.2. Probabilities for Braking and Steering Events

**Figure 5:**
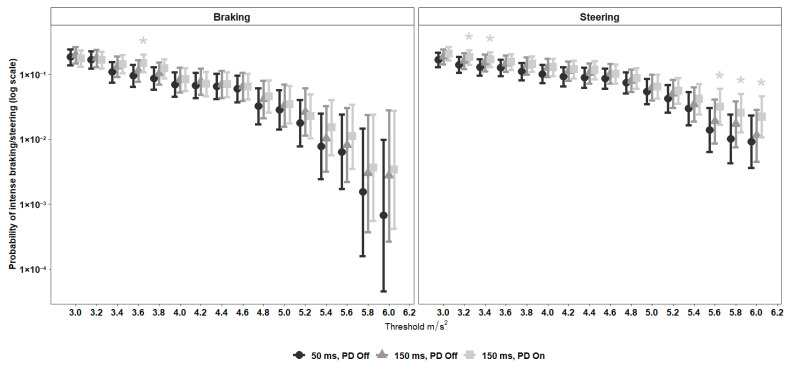
Estimated probability (log-scale) of intense braking (**left** panel) and steering (**right** panel) events as a function of acceleration threshold (2.0–6.0 m/s^2^) under three experimental conditions. Notes: (1) Asterisks (*) indicate a significant difference (*p* < 0.05) between the 150 ms delay + PD On condition and the baseline (50 ms delay + PD Off); no other pairwise contrasts reached significance. Thresholds are shown up to 6.0 m/s^2^; at higher values, there were too few intense events for the models to converge.

Figure 5 presents the probability of intense braking and steering based on the logistic regression model (Equation (2), Section 4). As expected, across all conditions, intense events were less frequent than mild ones, as indicated by the descending patterns along the x-axis. Overall, the PD condition showed a slightly higher probability of braking and steering events. For braking, probabilities under PD On were descriptively higher than those of PD Off across several mid-range thresholds, although none of these differences reached statistical significance. For steering, three out of five significant differences (marked with asterisks) appeared at the highest intensity thresholds, where the 150 ms PD On condition yielded a modestly higher probability of extreme steering events compared with the 50 ms PD Off baseline. The 150 ms PD Off condition did not differ significantly from the baseline at any threshold. In summary, the predictive display did not reduce the frequency of intense braking or steering and was associated with slightly higher probabilities of such events at selected thresholds.

#### 5.2.3. Maximal Braking and Steering Intensities by Driving Challenge

Two additional measures for driving safety that we compared between the experimental conditions were the maximal braking and maximal steering intensity, as stronger braking and steering events might point to evasive maneuvers from hazards that were detected late or not estimated correctly [39,41,42].

**Figure 6:**
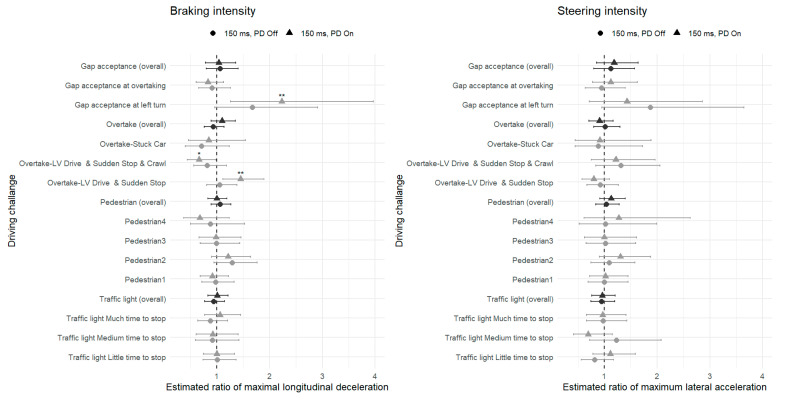
Forest plots of braking (**left** panels) and steering (**right** panels) intensity ratio (150 ms conditions vs. 50 ms delay baseline) by PD mode (● Off/▲ On), for each driving challenge (y-axis). Error bars show 95% CIs from the linear mixed-effects model. Asterisks denote model-based significance (** *p* < 0.01).

Figure 6 plots the ratio of maximal braking (left panel) and steering (right panel) intensities under 150 ms versus 50 ms delay, separately for PD Off (●) and On (▲), across the four challenge types. In the steering panel, aggregate ratios for traffic light, pedestrian, overtaking, and gap acceptance challenges cluster tightly around unity, with no significant deviation. At the individual challenge level, all confidence intervals include 1.0, and no segment shows a statistically significant change. For instance, gap acceptance at left turn (PD On) = 1.43 (CI: 0.72–2.86) and Overtake–LV Drive and Sudden Stop (PD On) = 0.80 (CI: 0.58–1.10). Overall, maximal steering responses remained stable under both delay and display conditions.

The braking panel shows no significant aggregate effects; all group-level ratios (pedestrian, traffic light, overtaking, and gap acceptance) remain close to 1.0, with confidence intervals spanning 1.0. At the challenge level, significant differences appear only under the 150 ms PD On condition. Gap acceptance at left turn shows a significant increase in maximal braking intensity (ratio = 2.23, CI: 1.26–3.96, *p* = 0.0061), as does Overtake–LV Drive and Sudden Stop (ratio = 1.45, CI: 1.11–1.89, *p* = 0.0060). In contrast, Overtake–LV Drive and Sudden Stop and Crawl exhibit a significant decrease (ratio = 0.66, CI: 0.44–0.98, *p* = 0.0404). All other segments show wide confidence intervals that include 1.0.

Taken together, these results demonstrate that maximal steering responses remained stable under both delay and display modes. The PD was associated with segment-specific changes in maximal braking, with stronger braking in the gap acceptance at left turn and Overtake–LV Drive and Sudden Stop challenges, and weaker braking in Overtake–LV Drive and Sudden Stop and Crawl, while aggregate measures remained non-significant.

### 5.3. Mental Workload

**Figure 7:**
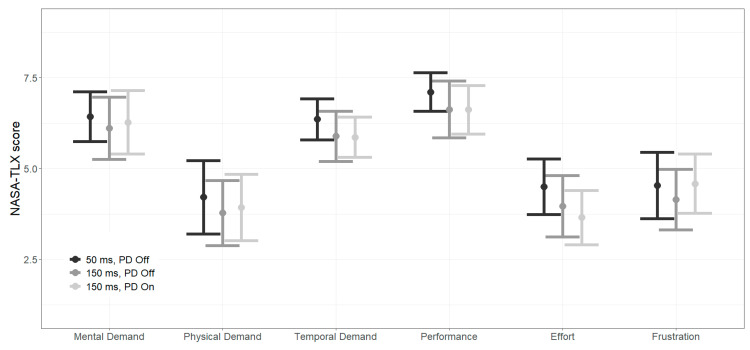
Participants’ mean scores (and CI’s) on the six subscales of the NASA-TLX according to the experimental conditions.

Figure 7 displays participants’ mean NASA-TLX scores and corresponding 95% confidence intervals across the three experimental conditions: 50 ms PD Off (black), 150 ms PD Off (dark gray), and 150 ms PD On (light gray). Considering that the confidence intervals across all three conditions overlapped substantially for every subscale, indicating that no differences reached statistical significance. In addition, a linear mixed-effects model that included the condition-by-subscale interaction (see Section 4) revealed no significant interaction terms, indicating that neither delay nor the PD altered workload across the NASA-TLX subscales, thus confirming the descriptive analysis in Figure 7.

### 5.4. Views About PD Usability

**Figure 8:**
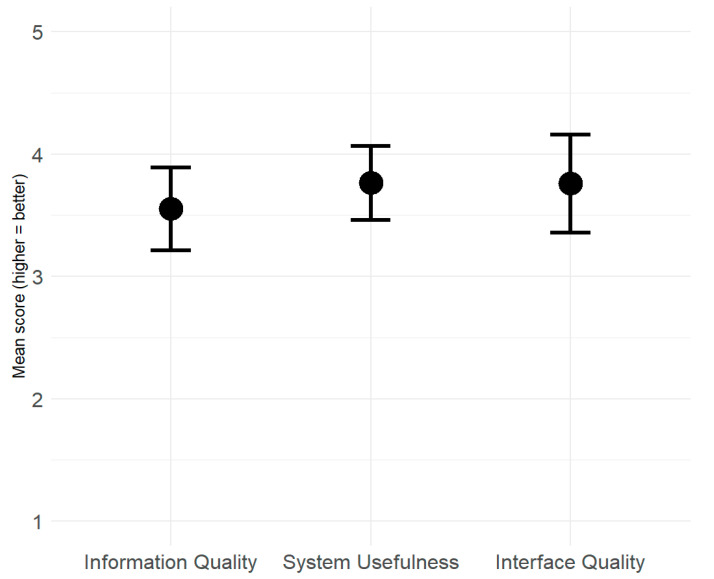
Means and 95% confidence intervals for participants’ ratings for the three PSSUQ subscales for the predictive display.

To assess users’ subjective impressions of the system, participants completed the PSSUQ following task completion. Figure 8 presents the mean scores and corresponding 95% confidence intervals for the three PSSUQ subscales: Information Quality, System Usefulness, and Interface Quality. Scores were given on a 5-point Likert scale, where higher values indicate more favorable perceptions.

Participants rated Information Quality with a mean of 3.52 (CI: 3.24–3.80), System Usefulness at 3.68 (CI: 3.39–3.97), and Interface Quality at 3.72 (CI: 3.43–4.01). These results indicate moderately positive usability perceptions across all three dimensions. Although Interface Quality showed the highest average score, the overlapping confidence intervals suggest no statistically reliable differences among the subscales.

## 6. Discussion

This study evaluated the influence of PDs on teleoperated driving performance, safety, and workload under a moderate 150 ms communication delay. Such time delay levels are typical of 4G and 5G networks [2,27] and thus represent realistic conditions for emerging teleoperation services. Participants drove in a simulated urban environment that included challenges such as pedestrian crossings, overtaking, and responding to changing traffic lights. The results across efficiency, safety, and mental workload indicate two main conclusions: first, the predictive display did not yield measurable improvements under moderate delay; second, human teleoperators were able to maintain stable control and workload even with a 150 ms delay, suggesting robust tolerance to realistic communication time delays.

### 6.1. Predictive Display Effect

Across all measures, the PD did not produce statistically significant improvements in performance or safety. Completion times, navigation errors, and crash rates were comparable across conditions. The PD did not significantly change task completion times (Figure 4), and navigation errors were rare and evenly distributed (three, five, and two errors for the 50 ms, 150 ms without PD, and 150 ms with PD, respectively). Crash rates were low across all conditions (three events under each 150 ms delay and none at 50 ms).

Analysis of the probability of intense driving events confirmed this pattern (Figure 5). The probability of intense braking or steering events followed the expected descending trend with increasing threshold, and differences between conditions were generally small. For braking, the PD condition showed a modest descriptive tendency toward higher probabilities across several thresholds, yet with confidence interval overlap and only one isolated significant difference. For steering, significant differences appeared at the highest intensity thresholds, where the PD condition yielded slightly higher probabilities of extreme steering events.

Figure 6 further shows that maximal braking and steering intensities remained statistically unchanged between conditions, with localized but mixed differences: higher braking in the gap acceptance at left turn and Overtake LV Drive and Sudden Stop challenges, and lower braking in Overtake LV Drive and Sudden Stop and Crawl. Overall, the PD neither improved nor degraded vehicle control or stability, and completion time measures were effectively stable across delay and display modes. Mixed results appeared in Graf et al. [29] who examined lane changes performance with and without a predictive corridor (an aid combining PD with location estimation if communication was interrupted) and found that during double-lane change (similar to overtaking), participants had less cons along the sides of lane but deviate (steer) more prominently with the mitigation aid showing significantly higher root square mean error especially on the second lane change when returning to the original lane.

Since the tested time delay levels did not seriously impair operators’ capacity to maintain control (see elaboration in Section 6.2), the potential for further improvement within the typical 5G networks appears limited. The additional predictive element may therefore have introduced minor visual redundancy rather than meaningful compensation. Previous research similarly indicates that PD are most beneficial under higher or fluctuating delays, typically between 500 and 1000 ms [20,21,26]. The present findings thus help define the boundary of PD usefulness: within moderate, stable delays, human operators can maintain performance without assistance.

The functional value of a PD may depend on the underlying control demands. Predictive visualization aligns naturally with continuous control tasks, those governed by feedback loops and smooth trajectory maintenance (see the Neumeier et al. [25] study), where it can help the operator minimize phase lag and improve tracking stability. By contrast, in discrete decision-making tasks, such as deciding whether to stop (traffic light challenge), yield (pedestrian or gap acceptance challenge), or commit an action (overtake challenge), the predictive information may add little or increase perception. For instance, in the gap acceptance with the left turn challenge, participants often bring the vehicle to a complete stop before turning. Under these zero-speed conditions, the PD overlay therefore disappears because the prediction term (delay × speed) equals zero. This temporary absence of the predictive cue may have made participants hesitant to initiate movement, thereby extending completion times (Figure 5) or requiring them to brake harder (Figure 6) when the PD reappears during the acceleration to turn, suggesting that the vehicle was positioned further than intended.

### 6.2. Human Tolerance to Moderate Time Delay

The present results showed that operator performance and workload at a 150 ms delay without the PD were largely comparable to those at 50 ms. Statistical analyses revealed no significant differences in completion time, crash rate, or NASA-TLX workload between these two time delay levels. Workload subscale means differed by less than half a point on the 1–9 scale (Figure 7), supporting the conclusion that a moderate time delay did not impose substantial additional cognitive or motor demands.

At the same time, some performance patterns suggest adjustments to the delay. Both braking and steering intensities remained largely stable under the 150 ms delay, showing only small, non-significant (Figure 5) and non-systematic variations across challenges (Figure 6). The broader literature aligns with this interpretation. Musicant, Botzer, and Shoval [14] found that teleoperators maintained stable performance and workload when delay increased from 50 to 150 ms in highway driving, with significant degradation only at 250 ms. Although their driving context differed from the present urban setting, the pattern suggests that operators can successfully adapt to delays of this size. Neumeier et al. [25] similarly observed a gradual decline with increasing time delay but reported statistical significance only at larger round-trip delays near 300 ms.

Taken together, these studies and the current findings indicate that the delay level tested lies near the lower boundary where teleoperation performance begins to show measurable degradation. Contemporary teleoperation services based on 4G and 5G connectivity often achieve sub-200 ms delays, implying that additional compensation mechanisms may add little functional value under normal operational conditions [2].

### 6.3. Usability and Implications for System Design

Although PDs did not produce measurable behavioral or workload improvements, they were positively received. Participants rated information quality, system usefulness, and interface quality at mean values of 3.5–3.7 on the 1–5 PSSUQ scale. Lewis [43] conducted psychometric evaluation of the PSSUQ by analyzing data from prior usability studies. Depending on the version of the PSSUQ, the overall scale means across studies ranged from 2.82 to 3.61 on a 1–7 scale. Hence, our participants’ ratings on a 1-to-5 scale can be considered moderately favorable, indicating that the display was clear and acceptable to use, even if it did not enhance objective performance.

### 6.4. Limitations

Several limitations should be acknowledged. First, the study used a driving simulator, which may not fully capture the attentional and emotional demands of real-world teleoperation. Real-world teleoperators experience environmental uncertainty, multitasking, and communication fluctuations that may or alter the role of PDs. Second, the sample consisted of university students rather than professional operators. Their limited teleoperation experience may have affected control strategies and the ability to benefit from the PD benefits. The sample sizes in the PD studies that we reviewed (Table 1) ranged between 5 [27] and 57 participants [21]. The sample size of the present study is comparable to prior work but remains modest. Our sample is relatively skewed toward female participants (21 of 29); we note that Neumeier et al. [25] had the opposite participant mix (6 females out of 28) and reported similar outcomes regarding the insignificant effect of moderate time delay on deceleration intensities. Third, crash and navigation error rates were low, limiting statistical power to detect differences in rare events. Finally, this study evaluated one PD implementation. Different visual formats, levels of transparency, or predictive horizons might yield different effects. Future analyses of these data may reveal underlying physiological correlates that complement the behavioral and subjective findings.

### 6.5. Summary

Across a comprehensive set of performance, safety, workload, and usability measures, the findings converge on two central conclusions. First, PDs did not significantly affect efficiency, safety, or workload under a moderate 150 ms delay. The predictive information appeared redundant when drivers could naturally compensate for modest feedback delays. Second, human teleoperation performance remained stable across delay conditions, suggesting that moderate time delay, typical of current networks, does not impair safety, efficiency, or workload. Considering findings from the studies we reviewed (see Table 1), the benefits of PDs lie in communication time delays exceeding 150 ms, which are less typical in 4G/5G networks, or in those that exhibit a prominent variance component [20].

### 6.6. Future Directions

Future research should include experienced teleoperators and incorporate real-world or semi-naturalistic driving tasks. Studies should examine how performance with PD evolves over longer durations and in multitasking contexts where operator fatigue and distraction are more likely. Eye-tracking (e.g., [29]) and physiological data can enhance understanding of cognitive load and visual strategies associated with PD use. Additionally, alternative PD formats, such as rendering the received image by projecting the surrounding [27], should also be evaluated under the same conditions as in the current study. Finally, future work can explore PD performance under degraded or variable communication conditions and in combination with other driver-assistance technologies to assess their combined potential in complex teleoperation environments.

## Figures and Tables

**Table 1:** Studies on predictive display.

Study Aim (Number of Participants)	(D) Delay (ms) (S) Scenario Characteristics (O) Other Road Users	Task	Results
Brudnak [26]: Develop and test a PD in simulation for N = 5 simulation runs for each experimental condition: no delay, delay, and delay + PD.	D—500 (fixed) S—Simulated rural course O—No	Lane-following under speed constraints at turns and straight segments	In comparison to no delay: Without PD: −30% mean speed, +148% deviation, +180% heading error. With PD: −9% mean speed. 60% deviation, 62% heading error.
Prakash et al. [27]: Develop and assess the usefulness of a PD (N = 5) in three conditions: Real driving, teledriving, and teledriving with PD.	D—70–150 (variable) S—Real urban O—No	Cornering and double-lane changes	PD reduced path deviation, improved control, and driver confidence under realistic variable delay.
Dybvik et al. [21]: Assess the performance and workload in three conditions: no delay, delay, and delay + PD. (N = 57)	D—700 (fixed) S—Real ground remote vehicle O—No	Peg-in-hole precision navigation	PD significantly improved objective and subjective task performance by 20% and 14%. And reduced frustration by 11%—not statistically significant.
Davis et al., [20] Assess the effects of both fixed and variable time lag on a simulated driving in three conditions: no delay, delay, and delay + PD. (N = 10)	D—700 (fixed or variable) S—Simulated rural course O—No	Tight turns and slaloms	PD significantly reduced the average lane offset and increased vehicle speeds for both fixed and variable time lag. Subjective NASA-TLX scores indicate lower mental, temporal, effort, and frustration ratings.
Sharma & Rajamani [28]: Assess the effects of a PD with three conditions: no delay, delay, and delay + PD. (N = 5)	D—500 (fixed) S—Simulated curved road with 4 lanes O—Lead vehicle following task	Participants had to stay in their lane, maintain 30–35 mph, and follow a lead vehicle ahead.	The maximum lane deviation time was lower with PD than under the delay condition. The average speed was higher with PD (like the no delay condition) than under the delay condition
Graf et al. [29] The study examined the benefits of a predictive corridor (PC) interface, that combined PD and vehicle position prediction in case the ego vehicle automatically stops due to communication loss (N = 32).	D—400 (fixed) S—Simulated emergency break and lane change O—No	Two tasks—Emergency braking and lane change, with a 15km/h posted speed limit.	Cognitive Load: subjective NASA TLX scores and objective forehead and nasal temperature indicated less workload with PC. Performance: During lane changes, PC led to fewer cone hits (M = 0.74 SE = 0.4) and smaller deviation from the optimal path (RSME = 0.09 m, SE = 0.05). In stop-line braking, participants stopped closer to the line without PC (M = 0.56 m, SE = 0.24).

**Table 2:** Driving challenges description.

Challenge	Green Track (A)	Blue Track (B)	Purple Track (C)
	Number in the Figure—Distance from the Start Point		
**Pedestrian crossing** **(four variants)**	P.1–205 m Ego car turns right, and a pedestrian emerges from the left. 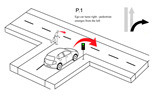 P.2–505 m 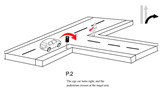 The ego car turns right, and the pedestrian crosses at the target arm.	P.3–145 m 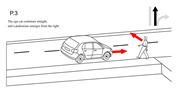 The ego car continues straight, and a pedestrian emerges from the right.	P.4–1186 m Ego car turns right, and a pedestrian emerges from the right. 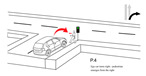
**Overtaking** The participant encounters an obstacle (Lead Vehicle—LV) that requires them to overtake. Three types: -LV Drive and Sudden Stop -LV Drive and Sudden Stop and Crawl -Stuck Car	Ov.1–665 m LV Drive and Sudden Stop: The LV drove at 10 m/s before a sudden stop (≈−8 m/s^2^)	Ov.2–373 m LV Drive and Sudden Stop and Crawl: The LV drove at 10 m/s before a sudden stop (≈−8 m/s^2^), and 4 s later resumed slowly at 5 m/s	Ov.3–445 m LV Drive and Sudden Stop: (as in Ov.1) Ov.4–1435 m Stuck Car: Stopped vehicle in the middle of the road, requiring the participant to overtake the obstacle.
	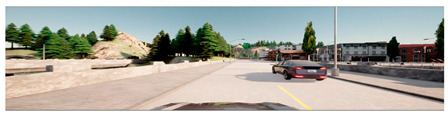		
**Gap Acceptance** Vehicles are approaching from the opposite direction in the oncoming lane at constant time gaps from each other. (Two variants: Overtake and left turn)	G.1–900 m Overtake an obstacle with oncoming traffic. The first 10 vehicles approach with a 15 s gap and an additional 5 vehicles approach with an 18 s gap. 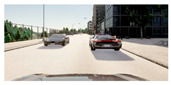		G.3–770 m Turn left with oncoming traffic. The first 10 vehicles approach with an 8 s gap and an additional 5 vehicles approach with a 12 s gap. 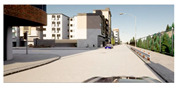
**Traffic light** The traffic light turns yellow for 3 s according to three variants: Little (2 s), Medium (3 s) and Much (5 s) time to reach the traffic light at the onset of the yellow light	T.1–1175 m Little time(s) M = 2, SD = 0.3 s	T.2–685 m Much time: M = 5 s, SD = 0.8 s T.1–1175 m Medium time: M = 3 s, SD = 0.02 s	T.3–154 m Much time: M = 5 s, SD = 1.6 s T.1–1735 m Little time: M = 2 s, SD = 0.3 s
	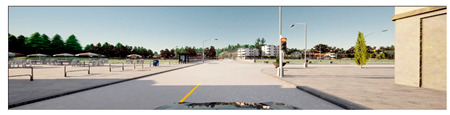		

## Data Availability

Data is unavailable due to privacy or ethical restrictions.
